# Brief Intervention in Type 1 diabetes – Education for Self-efficacy (BITES): Protocol for a randomised control trial to assess biophysical and psychological effectiveness

**DOI:** 10.1186/1472-6823-7-6

**Published:** 2007-09-14

**Authors:** Jyothis T George, Abel Peña Valdovinos, Jonathan C Thow, Ian Russell, Paul Dromgoole, Sarah Lomax, David J Torgerson, Tony Wells

**Affiliations:** 1 Dept. of Diabetes, York Hospital, York, UK; 2 División de Innovación en Servicios de Salud, Coordinación de Políticas de Salud, Dirección de Prestaciones Médicas, Instituto Mexicano del Seguro Social, Mexico; 3 University of Wales, Bangor, UK; 4 Independent Nurse Practitioner, York, UK; 5 York Trials Unit, University of York, York, UK; 6 Dept. of Psychology, City Hospitals, Sunderland, UK

## Abstract

**Background:**

Self management is the cornerstone of effective preventive care in diabetes. Educational interventions that provide self-management skills for people with diabetes have been shown to reduce blood glucose concentrations. This in turn has the potential to reduce rates of complications. However, evidence to support type, quantity, setting and mode of delivery of self-management education is sparse.

Objectives: To study the biophysical and psychological effectiveness of a brief psycho-educational intervention for type 1 diabetes in adults.

**Methods/Design:**

Design: Randomised controlled clinical trial.

Setting: Multidisciplinary specialist diabetes centre.

Hypothesis: Our hypothesis was that the brief (2.5-day) intervention would be biophysically and psychologically effective for people with type 1 diabetes.

Intervention: A brief psycho-educational intervention for type 1 diabetes developed by a multi-professional team comprising of a consultant diabetologist, a diabetes specialist nurse, a specialist diabetes dietician and a clinical health psychologist and delivered in 20 hours over 2.5 days.

Primary outcomes: HbA1c and severe hypoglycaemia.

Secondary outcomes: Blood pressure, weight, height, lipid profile and composite psychometric scales.

Participants: We shall consent and recruit 120 subjects with postal invitations sent to eligible participants. Volunteers are to be seen at randomisation clinics where independent researcher verify eligibility and obtain consent. We shall randomise 60 to BITES and 60 to standard care.

Eligibility Criteria: Type 1 diabetes for longer than 12 months, multiple injection therapy for at least two months, minimum age of 18 and ability to read and write.

Randomisation: An independent evaluator to block randomise (block-size = 6), to intervention or control groups using sealed envelopes in strict ascendant order. Control group will receive standard care.

Assessment: Participants in both groups would attend unblinded assessments at baseline, 3, 6 and 12 months, in addition to their usual care. After the intervention, usual care would be provided.

Ethics approval: York Research Ethics Committee (Ref: 01/08/016) approved the study protocol.

**Discussion:**

We hope the trial will demonstrate feasibility of a pragmatic randomised trial of BITES and help quantify therapeutic effect. A follow up multi-centre trial powered to detect this effect could provide further evidence.

**Trial registration:**

Current Controlled Trials ISRCTN75807800

## Background

Self-management is a critical component in effective preventive care for people with diabetes [1,2] and a key focus area for public policy in the UK. [3] Educational interventions designed to provide diabetes patients with self-management skills have been shown to reduce blood glucose concentration [4-7] which in turn has the potential to reduce rates of complications [8]. However, the evidence to support type, quantity, setting and mode of delivery of self-management education is sparse.

In the UK, the Dose Adjustment for Normal Eating (DAFNE) group was the first to deliver an effective educational programme for type 1 diabetes [5]. DAFNE is based on a successful inpatient programme developed in Düsseldorf, Germany [4] and is delivered in 35 hours over five consecutive days. Many diabetes teams in the UK have since developed brief educational programs delivered in 15 to 24 hours over 4 to 6 weeks [9].

In response to Department of Health initiatives [10] and in light of reported benefits in trials [4,6], we developed an educational and psychosocial intervention that is acceptable to people with diabetes, feasible and cost-effective in practice. This local study will also test the feasibility of a multi-centre pragmatic randomised trial in this field, and provide evidence about the likely size of the therapeutic effect.

## Methods/Design

### Setting

A randomised pragmatic controlled clinical trial at our multidisciplinary specialist diabetes centre.

### Intervention

The course (Brief Intervention in Type 1 diabetes: Education for Self-efficacy -BITES) was developed by a multi-professional team comprising a Consultant Diabetologist, a Diabetes Specialist Nurse, a Specialist Diabetes Dietician and a Clinical Health Psychologist; and delivered as a 2.5-day course over a 6-week period to allow participants time to practice and reflect between sessions. A Diabetes Specialist Nurse and Specialist Diabetes Dietician facilitated sessions.

Based on adult learning principles, BITES includes use of cognitive behavioural techniques. Coping, control and strategies for prioritising are explored. Imaginative insulin use, carbohydrate estimation, glycaemic index as well as pre-and post-meal blood glucose monitoring are promoted. Participants are encouraged to self-adjust insulin doses according to their carbohydrate intake and level of activity. Changing negative to positive thoughts and maintaining changes are discussed. Participants are introduced to a fictitious individual with diabetes whom they mentored throughout the course and discuss helping them around the change cycle. [11] Home reflection and sharing are components of the course rather than simple provision of information. The time-table of the course outlining its contents is provided in table 1

**Table 1:** Timetable for the Educational Intervention

**Day 1 in Week 1** (0900–1600)	**Day 2 in Week 2** (0900–1600)	**Day 3 in Week 6** (0900–1300)
1 Introduction.	6 Feedback from last session & Workbook.	11 Feedback from last session & Workbook.
2 Freedom and control in day to day diabetes.	7 Using insulin as a tool.	12 Fine-tuning – activity, eating out & illness.
3 Principles of healthy eating.	8 Understanding insulin adjustment.	13 "Going for Gold" (Motivational video).
4 Analysing food content & estimating carbohydrates.	9 Blood glucose monitoring as a tool (including testing before & after meals).	14 Maintaining change.
5 Group exercise – analysing food content.	10 Exercise in "free diet" – combined food analysis and insulin adjustment.	
**Workbook**	**Workbook**	
Food analysis – practical exercises & daily comments.	Food analysis & insulin adjustment – daily comments.	
1 scheduled phone support call	1 scheduled phone support call	

### Participants and protocol

Participants are recruited from those attending a single specialist diabetes service in a hospital setting. Eligibility criteria: Type 1 diabetes for longer than 12 months, multiple injection therapy for at least two months, minimum age of 18 and ability to read and write. York Research Ethics Committee (Ref: 01/08/016) approved the study protocol.

**Figure 1:**
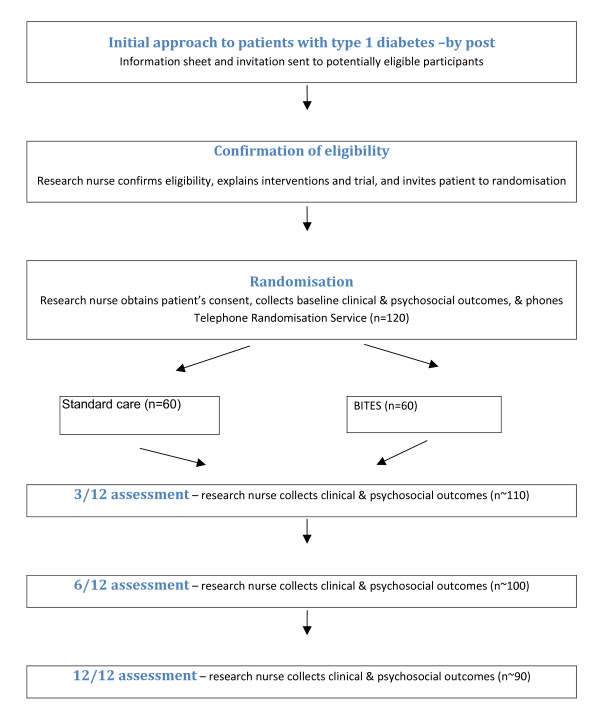
Proposed flow of volunteers in BITES study.

Postal invitations are sent to eligible participants along with information about the study. Willing respondents are seen at randomisation clinics where the research team verified eligibility and obtained written informed consent. An independent evaluator then allocates participants using block randomisation (block size = 6), to intervention or control groups using sealed envelopes in strict ascendant order. Intervention group is offered the course in six groups of 8 to 10. Participants in both groups attend four unblinded assessments at baseline, 3, 6 and 12 months, plus their usual care. After the course, standard care would continue. The proposed flow of volunteers in the trial is outlined in figure 1.

The control group is seen in their usual diabetes clinic in addition to their study appointments. Patients had open access to Diabetes Specialist Nurses and Specialist Diabetes Dietician, and access to the Clinical Health Psychologist by referral. Information covered in the course is not withheld from individuals in the control group if requested or deemed appropriate to their care. Control group receives the full course 12 months later.

### Primary outcomes

HbA1c is measured (DCCT aligned) and severe hypoglycaemia recorded at baseline, 3, 6 and 12 months. Severe hypoglycaemia is defined as a recorded episode in which the patient required assistance with treatment and either documented blood glucose fell below 2.7 mmol/l or detected clinical signs that required oral carbohydrate administered by a third party, subcutaneous glucagon or intravenous glucose.

### Secondary outcomes

Blood pressure, weight, height (and calculated BMI in kg/m2), lipid profile and the number of daily insulin injections are used as secondary endpoints. Participants are asked to complete a psychosocial and knowledge questionnaire (221 items) with Cronbach alpha between 0.60 to 0.94. The questionnaire includes the following scales: MOS 36-Item Short-form Health Survey [12]; Illness Perception Questionnaire (IPQ) [13], Diabetes Knowledge Test (DKT) [14]; Diabetes Empowerment Scale (DES) [15]; Diabetes Treatment Satisfaction Questionnaire (DTS-Q) [16]; Hypoglycaemia Fear Scale (HFS) [17] and Diabetes Health Profile (DHP) [18].

### Statistical Analysis

We would use intention to treat analysis of covariance (ANCOVA) adjusting for baseline scores. To have 80% power to detect a 1% difference in HbA1c levels, which we deemed to be of clinical significance, requires us to recruit at least 90 participants. A P Value of < 0.05 is considered statistically significant.

### Technical Data

HbA1c (DCCT Aligned) is estimated by an ion-exchange high-performance liquid chromatography method [19] [Tosoh Medics, Foster City, California]. The normal range for healthy subjects (mean + 2 standard deviations) is 4.4 to 6.1%. Blood pressure, weight and height (and calculated BMI in kg/m2) are measured by standardised methods.

### Economic analysis

To evaluate BITES we shall use incremental cost-utility analysis from the perspective of both the NHS and society in general. To estimate the utility of changes in health status we shall use the EuroQoL, a generic measure of health purposefully developed to generate a cardinal index of health, thus giving it considerable potential for use in economic evaluation. [20]

## Discussion

There are up to 20 different brief interventions developed and used in different parts of the UK [9], with recommendations to offer such interventions to all patients with diabetes [2]. Educational theory suggests efforts to change behaviour are more likely to be successful in the long term where information is 'drip fed' rather than being provided intensively in a short space of time [21] and hence our course, like other brief interventions [9], is delivered over few weeks. We hope the trial will confirm the feasibility of a multi-centre pragmatic randomised trial of BITES. If so, it will also provide evidence about the likely size of the therapeutic effect. This will enable us to make the national trial large enough to yield adequate statistical power to detect such an effect across many centres.

To disseminate the findings of the single-centre trial, we shall submit up to three papers for peer-reviewed publication. The first will report our multi-disciplinary evaluation of BITES, ideally in a general medical journal. The second will describe BITES in more detail, preferably in a specialist diabetes journal. The third will report our psychometric analysis of our portfolio of eight patient-assessed outcome measures, preferably in a specialist health services research journal.

## Abbreviations

BITES, Brief Intervention in Type 1 Diabetes – Education for Self-Efficacy; BMI, Body Mass Index; DCCT, Diabetes Complications Control Trial; HbA1c, Haemoglobin A1C; UK, United Kingdom of Great Britain and Northern Ireland

## Competing interests

The author(s) declare that they have no competing interests.

## Authors' contributions

AP, JG and JT undertook the literature review for this paper and were responsible for drafting this manuscript. JT, IR and DT identified the research question and developed the initial research protocol and educational intervention along with SL, AP, TW and PD. AP and JG were involved in analysis and interpretation of data. All authors have been involved in drafting the manuscript or revising it critically for intellectual content and have given their final approval.

## Pre-publication history

The pre-publication history for this paper can be accessed here:



## References

[B1] Mensing C, Boucher J, Cypress M, Weinger K, Mulcahy K, Barta P, Hosey G, Kopher W, Lasichak A, Lamb B, Mangan M, Norman J, Tanja J, Yauk L, Wisdom K, Adams C (2007). National standards for diabetes self-management education. Diabetes Care.

[B2] National Institute of Clinical Excellence (2003). Technology appraisal guidance 60. Guidance on the use of patient education models for diabetes. London.

[B3] Deparment of Health (2003). National service framework for diabetes: Standards. London.

[B4] Muhlhauser I, Bruckner I, Berger M, Cheta D, Jorgens V, Ionescu-Tirgoviste C, Scholz V, Mincu I (1987). Evaluation of an intensified insulin treatment and teaching programme as routine management of type 1 (insulin-dependent) diabetes. The bucharest-dusseldorf study. Diabetologia.

[B5] DAFNE Study Group (2002). Training in flexible, intensive insulin management to enable dietary freedom in people with type 1 diabetes: Dose adjustment for normal eating (DAFNE) randomised controlled trial. BMJ.

[B6] Pieber TR, Brunner GA, Schnedl WJ, Schattenberg S, Kaufmann P, Krejs GJ (1995). Evaluation of a structured outpatient group education program for intensive insulin therapy. Diabetes Care.

[B7] Samann A, Muhlhauser I, Bender R, Kloos C, Muller UA (2005). Glycaemic control and severe hypoglycaemia following training in flexible, intensive insulin therapy to enable dietary freedom in people with type 1 diabetes: A prospective implementation study. Diabetologia.

[B8] Lasker RD (1993). The diabetes control and complications trial – Implications for policy and practice. N Engl J Med.

[B9] Type 1 education network. http://www.diabetes.nhs.uk/downloads/Type_1_Education_Network.pdf.

[B10] Department of Health (2001). The Expert Patient: A New Approach to Chronic Disease Management for the 21st Century.

[B11] Prochaska JO, DiClemente CC (1983). Stages and processes of self-change of smoking: Toward an integrative model of change. J Consult Clin Psychol.

[B12] Ware JE, Sherbourne CD (1992). The MOS 36-item short-form health survey (SF-36). Conceptual framework and item selection. Med Care.

[B13] Moss-Morris R, Weinman J, Petrie K, Horne R, Cameron L, Buick D (2002). The revised illness perception questionnaire (IPQ-R). Psychology & Health.

[B14] Fitzgerald JT, Funnell MM, Hess GE, Barr PA, Anderson RM, Hiss RG, Davis WK (1998). The reliability and validity of a brief diabetes knowledge test. Diabetes Care.

[B15] Anderson R, Funnell M, Fitzgerald J, Marrero D (2000). The diabetes empowerment scale: A measure of psychosocial self-efficacy. Diabetes Care.

[B16] Lewis KS, Bradley C, Knight G, Boulton AJ, Ward JD (1988). A measure of treatment satisfaction designed specifically for people with insulin-dependent diabetes. Diabet Med.

[B17] Cox DJ, Irvine A, Gonder-Frederick L, Nowacek G, Butterfield J (1987). Fear of hypoglycemia: Quantification, validation, and utilization. Diabetes Care.

[B18] Goddijn P, Bilo H, Meadows K, Groenier K, Feskens E, Meyboom-de Jong B (1996). The validity and reliability of the diabetes health profile (DHP) in NIDDM patients referred for insulin therapy. Qual Life Res.

[B19] Khuu HM, Robinson CA, Goolsby K, Hardy RW, Konrad RJ (1999). Evaluation of a fully automated high-performance liquid chromatography assay for hemoglobin A1c. Arch Pathol Lab Med.

[B20] Brooks R (1996). EuroQol: The current state of play. Health Policy.

[B21] Reece I, Walker S (2000). Chapter 2. Teaching, Training and Learning: A Practical Guide.

